# The complete mitochondrial genome sequence and phylogenetic analysis of Alpine Merino sheep (*Ovis aries*)

**DOI:** 10.1080/23802359.2020.1720536

**Published:** 2020-02-03

**Authors:** Guoyan Qiao, Hao Zhang, Shaohua Zhu, Chao Yuan, Hongchang Zhao, Mei Han, Yaojing Yue, Bohui Yang

**Affiliations:** a Institute of Husbandry and Pharmaceutical Sciences, Chinese Academy of Agricultural Sciences, Lanzhou, People’s Republic of China;; b Sheep Breeding Engineering Technology Research Center of Chinese Academy of Agricultural Sciences, Lanzhou, China

**Keywords:** Mitochondrial genome, Alpine Merino sheep (*Ovis aries)*, phylogenetic analysis

## Abstract

Alpine Merino sheep is one of the most important fine-wool sheep breeds in China. In this study, we present the complete mitogenome of Alpine Merino sheep for the first time. The genome has a length of 16,619bp, containing 13 protein-coding genes, 2 ribosomal RNA genes, 22 transfer RNA genes, and a control region (D-loop). Phylogenetically, the Alpine Merino sheep is closer to Oula Tibetan sheep and Tashkurgan sheep. This report provided new data for the phylogeny of Alpine Merino sheep.

The Alpine Merino sheep is a fine-wool sheep breed that was developed in the high and cold Qilian mountainous pasture in Gansu province, China, where the altitude is between 2400 and 4070 m above sea level (Li and Purvis 2012). It has good adaptability to the ecological environment of cold and arid mountainous areas at high altitude, and has the remarkable characteristics of coarse feeding resistance, strong disease resistance and high survival rate of reproduction. The complete mitochondrial genome sequence of Alpine Merino sheep has not been reported.

Samples of this work were collected from Gansu Province Sheep Breeding Technology Extension Station, Sunan County, Gansu Province, China (N38°83′, E99°62′). Blood samples were frozen on the first floor of the −80 °C refrigerator (NO.3) in sample storage room of the Genetic and Breeding Laboratory of Sheep Breeding Engineering Technology Research Center of Chinese Academy of Agricultural Sciences. The storage room number is r704.Total genomic DNA was extracted from the blood samples using Easy Pure Blood Genomic DNA Kit (Transgen Biotch, Beijing, China) according to manufacturer’s instructions. The DNA samples were frozen on the third floor of the −20 °C refrigerator in the same sample storage room. The refrigerator number is NO. 1. The DNA samples numbers were 5AM191012D, 5AM191013D, 5AM191015D. The complete mitogenome of Alpine Merino sheep was determined using polymerase chain reaction based on 11 pairs of primers and sequencing (Guo et al. 2005) and conducted with the Illumina HiSeq X™ Ten Sequencing System (Illumina, CA, USA) by Annoroad Gene Technology (Beijing, China). The sequencing results were assembled using DNASTAR5.0 software. The complete sequence and annotation were submitted and deposited in GenBank with an accession number MN882069 (Figure 1).

**Figure 1.**
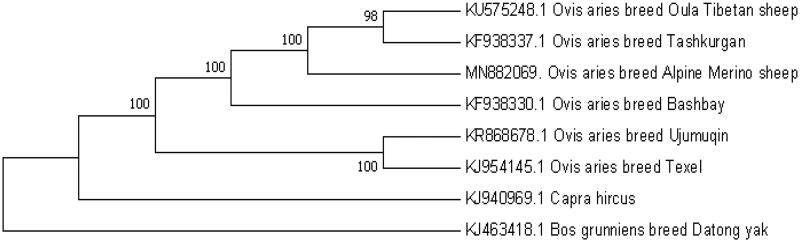
Phylogenetic tree based on the mitochondrial genome of six species using the neighbor-joining (NJ) method of 1000 bootstrap replicates..

The whole mitogenome is a circular DNA molecule of 16,619bp, consisting of 13 protein-coding genes (ND1-6, ND4L, COX1-3, CYTB, ATP6, and ATP8), 22 transfer RNA genes, 2 ribosomal RNA genes (12 s and 16 s ribosomal RNA), and a control region(D-loop). The gene order and composition of the mitochondrial genome of Alpine Merino sheep was similar to that of other sheep species. The ratio of basic nucleotide composition is: 33.67% for A, 13.11% for G, 27.38% for T, and 25.84% for C. The C + G base composition (38.94%) was lower than A + T (61.06).

To ascertain the phylogenetic placement of Alpine Merino sheep, a neighbor-joining phylogenetic tree was constructed with MEGA7.0 software (Kumar et al. 2016), with 1000 bootstrap replicates, using the coding sequences of 5 other Ovis species and 1 Caprinae species, using the yak(Bos mutus) as an outgroup. According to phylogenetic tree analysis, Alpine Merino sheep has a closer genetic relationship with Oula Tibetan sheep and Tashkurgan sheep. In summary, the complete mitochondrial genome sequence of Alpine Merino sheep can be further used for phylogenetic reconstruction and conservation strategies of the endemic species.
